# Protective factors in resilient South African youth with type 1 diabetes: A qualitative study

**DOI:** 10.4102/phcfm.v17i1.4935

**Published:** 2025-09-03

**Authors:** Simphiwe Mabizela, Elmari Deacon, Esme van Rensburg, Christiaan I. Bekker

**Affiliations:** 1 COMPRES Research Focus Area, Faculty of Health Sciences, North-West University, Potchefstroom, South Africa; 2 Optentia Research Unit, Faculty of Humanities, North-West University, Potchefstroom, South Africa

**Keywords:** adolescents, protective factors, qualitative descriptive design, resilience, self-management, type 1 diabetes, well-controlled

## Abstract

**Background:**

Self-managing type 1 diabetes (T1D) can be challenging, especially for adolescents in a critical developmental stage. Some adolescents struggle to successfully self-manage T1D and struggle to keep it well-controlled into adulthood. Despite this concern, there is a notable lack of evidence-based research on protective factors and/or resources to support adolescents living with T1D in South Africa.

**Aim:**

This study aimed to explore and describe the protective factors of resilience among South African adolescents living with well-controlled T1D.

**Setting:**

The study was conducted at the Centre for Diabetes and Endocrinology (CDE) in Parktown, Gauteng, South Africa, a specialised facility offering comprehensive, multidisciplinary care for adolescents with T1D.

**Methods:**

A qualitative descriptive research design was used, and seven semi-structured interviews were conducted, transcribed and thematically analysed.

**Results:**

Four themes emerged from the data: Just do it: Commit to the diabetes care plan; It takes a village to raise a child living with diabetes; The silver lining of developing positive characteristics and It’s a process of continuously learning about T1D.

**Conclusion:**

Internal abilities (planning, taking responsibility, perseverance and determinism) and external resources (parents, family members, school, mentors) foster positive outcomes and adjustment for adolescents with well-controlled T1D. The potential for adolescents with T1D to use available internal abilities and external resources in managing their diabetes could be beneficial to the successful management of T1D.

**Contribution:**

The study addressed a gap in understanding protective factors involved in the successful self-management of adolescents living with well-controlled T1D in South Africa.

## Introduction

Research on adolescents living with type 1 diabetes (T1D) often emphasises risk factors and poor outcomes, such as diminished well-being, poor glycaemic control and parental stress or depression.^1,2^ The concept of ‘risk’ broadly encompasses vulnerability to adverse outcomes like poverty, violence, inadequate care and emotional distress.^3^ Much of the literature highlights challenges in self-managing T1D^4^ and focuses more on barriers than on factors that promote positive adaptation.^5^ In contrast, protective factors – which reduce the likelihood of adverse outcomes in the face of adversity^6^ – have gained increasing attention. These include social support, a sense of belonging, access to healthcare and education, adaptive coping and self-worth.^6,7,8^ Such factors are associated with improved diabetes outcomes, including glycaemic control,^9,10,11^ enhanced self-management and quality of life.^12^ Resilience literature underscores the fact that protective factors can buffer against adversity and foster positive adjustment.^10,11,12^ Strengthening these resources can empower individuals and communities to cope more effectively with chronic conditions like T1D.^12^

Type 1 diabetes is a chronic autoimmune disease in which the immune system mistakenly attacks the pancreatic β-cells, resulting in insulin deficiency.^13,14^ Although commonly diagnosed in childhood or adolescence, it can occur at any age.^14^ In South Africa, diabetes mellitus is the leading cause of death among non-communicable diseases,^15^ with T1D prevalence nearly tripling from 2010 to 2019.^16^ Yet, many individuals struggle with glycaemic control,^16,17^ which is particularly demanding for adolescents who must manage insulin, blood glucose levels and diet rigorously.^18,19^ Exogenous insulin is necessary as part of a comprehensive care strategy for managing hyperglycaemia to prevent ketoacidosis.^20^ Therefore, the adolescent must actively participate in their treatment, thus making it a lifelong task.

Adolescence, defined as the developmental stage between 12 years and 18 years of age, involves the formation of identity, increased autonomy and intense social and emotional changes.^21,22^ Managing T1D during this period adds complexity, as adolescents face psychosocial challenges – including loneliness, depression and anxiety – while navigating disease self-management.^17,23^ This dual burden can lead to poor outcomes, but some adolescents nonetheless achieve reasonable control and demonstrate resilience. Understanding the protective factors that support this success is critical.

Resilience is the dynamic capacity to adapt to significant adversity^8^ and typically involves three elements: experiencing adversity, positive adaptation and the presence of protective resources.^8^ This process perspective suggests that individuals can adjust positively over time by drawing on both internal strengths and external supports. When protective factors are present, adolescents may flourish even while facing chronic health conditions.^8,9,10,24^ Social support, emotion regulation, self-efficacy and a sense of meaning are known to enhance resilience.^24,25,26,27,28,29^ Coping strategies such as problem-solving and cognitive reappraisal further assist individuals in managing stress and adversity.^27^ Access to external resources, including education, healthcare and community support, also serves as a key protective factor.^24^ Spirituality and religion, for some, provide motivation and strength to face illness.^30^ These resources may enhance adherence to self-care routines and glycaemic control.^31^ Previous research on protective factors in T1D has largely been quantitative, identifying elements such as initiative and self-control in children,^4,32^ and self-worth and social support in adolescents.^33^ However, a deeper qualitative understanding of the most influential protective factors in adolescents remains limited.

Therefore, this study explored the protective factors that support South African adolescents living with well-controlled T1D. The first aim was to understand the risks faced by these adolescents and the protective mechanisms that contribute to resilience. The second was to identify specific protective factors that promote resilience and enable effective self-management. By doing so, the study aims to contribute to a broader understanding of resilience and protective factors in chronic illness, particularly within the South African context. Thus, the study aimed to answer the following research question: *What are the protective factors of South African adolescents living with well-controlled T1D?*

## Research methods and design

### Study design

An exploratory, qualitative design grounded in the social constructivist approach was employed in this study to gain in-depth descriptions of the protective factors of resilience among South African adolescents living with well-controlled T1D. This approach allowed the researcher to gain deeper insight into the subjective experiences of these adolescents living with T1D and navigating the adjustments that come with the adolescent developmental phase, as well as the self-management of T1D.^34^

### Setting

This study was conducted at the Centre for Diabetes and Endocrinology (CDE) in Parktown, Johannesburg, Gauteng Province, South Africa. The CDE is a private managed care organisation specialising in comprehensive, person-centred diabetes management, with a model of care emphasising individualised treatment plans tailored to each patient’s needs. The multidisciplinary team includes doctors and nurses with access to other health practitioners to effectively support patients in managing their condition. The facility serves children and adolescents with T1D from Johannesburg and surrounding areas. Patients come from various socioeconomic backgrounds, reflecting the urban and peri-urban communities of the Gauteng province.

### Study population, sample size and sampling

This study’s participants comprised South African adolescents between the ages of 12 years and 18 years with well-controlled T1D (HbA1C of 7.5% – 8% or less [59 mmol/mol – 64 mmol/mol] over the past 12 months^35,36^) attending the CDE. The only exclusion criterion was that participants with other chronic conditions were excluded to avoid it influencing their management of T1D. The sample size was determined by saturation of data when no new themes were uncovered in the last two interviews.

Prospective participants were invited to participate in the study using an information leaflet distributed by the CDE, Parktown. Purposive sampling, more specifically, criterion sampling, was employed to select participants for this study.^37^ Key criteria were that they had been diagnosed for at least a year; were willing and able to communicate in English and had access to a computer or smartphone and an internet connection for the semi-structured interviews as they were conducted virtually.

### Data collection

The first author (S.M.) conducted seven semi-structured interviews from September 2021 to December 2021. Given that the coronavirus disease 2019 (COVID-19) limited face-to-face interactions, online interviews were conducted using video conference platforms to ensure the safety of participants and researchers. Given their circumstances and environments, the participants could indicate a time that suited them best. Interviews lasted between 40 min and 60 min, while being recorded on the online video conference platform. The prompts that guided the semi-structured interviews were: (1) How long have you been diagnosed with T1D?; (2) How would you describe your experience with managing T1D?; (3) How do you self-manage T1D?; (4) What challenges do you experience regarding diabetes management?; (5) What personal characteristics help you manage T1D?; (6) What outside sources help you to self-manage T1D?; and (7) What and/or who has played the biggest role in helping you maintain well-controlled T1D?

With the help and guidance of diabetes management experts, the first author reviewed her notes to minimise the likelihood of internal bias. Interviews were transcribed and analysed as they took place.

### Data analysis

The data obtained from the transcribed interviews were thematically analysed, as delineated by Braun and Clarke.^38^ The first author immersed herself in the data while transcribing and re-reading once coding occurred. The transcripts were then reviewed by the second (E.D.) and fourth authors (C.I.B.) who served as co-coders, and alignment discussions occurred before determining the themes. The field notes were also analysed to bracket the first author’s possible biases. This was all done manually, and no computer software was utilised. The identified themes included internal abilities and external resources as protective factors, based on theory, which enable resilience and effective diabetes management for these adolescents.

### Trustworthiness

This study applied Maxwell’s strategies^39^: Descriptive validity was achieved by keeping the video recordings and notes made during data collection as evidence of what occurred during the interviews. Interpretive validity was established by the first author, who conducted her coding and utilised the second and fourth authors as co-coders to ensure that the interpretation of the participants’ narratives aligned with hers and to discuss the formulation of themes. Theoretical validity was achieved by providing a precise and detailed description of the methodology used, as well as by reflecting on the existing literature on T1D and resilience theory regarding protective factors and ensuring that the descriptions of participant experiences were accurate and credible. Evaluative validity was established by the first author, who kept written records of her preconceived notions and assumptions about the study and took field notes to reflect on her thoughts and feelings whilst collecting and analysing the data.^40^

### Ethical considerations

The study received approval from the Optentia Research Unit’s scientific committee before obtaining ethical clearance from the North-West University Health Research Ethics Committee (NWU-HREC) (No. NWU-00221-21-A1), South Africa. The participants in this study were under 18 years and living with T1D, which was classified as a medium-risk study. Participants and their caregivers received all the information, and the researchers emphasised that participating in the study was entirely voluntary. Participants were informed that they could withdraw from the research at any time. There were no consequences for participants who chose not to participate. Parental permission and adolescent consent forms were signed before the interviews took place via the video conference platform, with witnesses present for the parent, the adolescent and the independent person to ensure that participation remained voluntary and free from coercion. This was done to follow COVID-19 regulations, thus upholding the participants’ and researchers’ safety. The participants then scanned or took a picture of the consent forms and emailed them to the independent person. Participants could indicate their preferred time for the interviews, as they were conducted online at a time and place convenient for them. Anonymity and confidentiality were also ensured by assigning pseudonyms and storing the consent forms separately from recordings and the participant code list.

## Results

The final study sample is shown in Table 1. It consisted of three female (two Caucasian and one Indian) and four male (Caucasian) adolescents between the ages of 12 years and 17 years. The mean age at which they were diagnosed was 3.6 years. Pseudonyms were used to protect participants’ identities.

**TABLE 1:** Biographical information and pseudonyms of participants.

Pseudonyms	Age	Gender	Time since diagnosis
John	17	Male	7 years
Olivia	13	Female	2 years
Liam	14	Male	2 years
Inca	13	Female	1 year 6 months
Nathan	12	Male	4 years
Aiden	13	Male	6 years
Ivy	15	Female	5 years

After the thematic analysis was conducted, the following themes emerged from the data: ‘Just do it: Commit to the diabetes care plan’, ‘It takes a village to raise a child living with diabetes’, ‘The silver lining of developing positive characteristics’, and ‘It’s a process of continuously learning about T1D.’

### Theme 1: Just do it: Commit to the diabetes care plan

Participants reported that committing to a diabetes care plan enabled them to manage their diabetes well. Creating and adhering to a care plan also helped keep diabetes within a well-controlled range and increased their motivation to maintain diabetes management behaviours.

All participants in the study emphasised the importance of planning and being prepared to manage their diabetes effectively. Ivy said: ‘[*M*]ake sure that you’ve got all your supplies definitely helps make sure that you manage your diabetes better. Uhm, you’ve got to be prepared for like anything [*participant laughing*] that could go wrong’ (15, F) and Inca (added ‘[*J*]ust bring like a lot of snacky stuff for school’ (13, F). The participants further revealed that creating a schedule makes a well-controlled T1D manageable. Liam said: ‘I have my own little mind map that I worked out’ (14, M) adding that he has learned the ‘exact carb ratio’ for each of his preferred meals. Olivia said: ‘I control my portions in how I eat, and I take enough insulin to cover my portions’ (13, F). The participants indicated that they followed and prepared their plan from the moment they woke up to when they went to bed. Nathan mentioned when he checks his insulin levels: ‘[*I check it*] before I eat, after I eat and like during the day. So, it’s a lot of times’ (13, M).

Managing T1D for participants involved *staying active* to keep the condition well-controlled on a continual basis. Nathan expressed that: ‘[*E*]xercise helps a lot with my blood sugar’ and ‘keep(s) it nice and stable’ (13, M). Olivia mentioned, ‘I do swimming every week, so once a week I do swimming. It would be like 4 to 5 times a month’ (13, F). Participants further added that staying active and exercising also helped keep their minds off the reality of T1D. John mentioned that ‘…being active as I can coz I found that if I don’t do something it kinda gets depressing’ (17, M).

Participants realise that self-managing T1D falls on them and becomes their responsibility. Ivy indicated: ‘[*I*]t’s my responsibility except at night’ (15, F). It has been beneficial to undertake the responsibility for controlling T1D:

‘I’ve had to do a lot of stuff by myself and I’ve actually found it rewarding to figure it out. Its like oh okay that’s what I do and see that it actually works.’ (John, 17 years, male)

The participants further mentioned that taking that responsibility has also made it more normal for them. Inca expressed: ‘Uhm so now I like walk around with my patch just like flashing, I’ll inject in public’ (13, F). Managing T1D in public and taking on that responsibility helps participants realise this is their responsibility. Inca said: ‘I don’t really care anymore because, I mean it’s something I have to deal with’ (13, F).

Participants revealed that committing to a diabetes care plan requires perseverance and acknowledged that managing T1D is challenging:

‘Uh, it it’s also a full-time job to manage your diabetes. And sometimes it can be a lot to carry around and deal with on a daily basis.’ (Ivy, 15 years, female)

‘I know each morning I wake up I am going to have to inject then to eat something … I know I am going to inject once a day and waking up and knowing that, isn’t very pleasant.’ (Liam, 14 years, male)

However, Liam further explains the benefits: ‘[*S*]elf-managing would be harder to do but there have been days that I had to control it myself and I have done quite well on those days’ (14, M).

### Theme 2: It takes a village to raise a child living with diabetes

All participants expressed the benefits of social support from family, friends, their communities or other individuals with the condition, as they recognised that managing a chronic condition can be difficult and stressful. Participants welcomed social support from others, as it helped them maintain well-controlled T1D and fostered a sense of community.

Participants shared how parents and other family members supported them with diabetes-management tasks and also provided emotional support. Olivia mentioned: ‘… my gran sacrifices a lot to help me control at night … and my sister, she also helps me stay calm’ (13, F). All participants mentioned their parents, with Inca stating: ‘[*T*]hey’re probably my biggest supporters as well’ (13, F). Participants reported that their support extends beyond family and legal guardians, including peer support. Nathan stated: ‘I also have like my friends nudging me like how is your reading doing? Are you fine?’ (12, M). Ivy echoed this by saying: ‘It’s been really good that I have a friend group that’s quite supportive, and they look out for me’ (15, F). Some participants were grateful for external support from the school. Olivia mentioned that: ‘[*A*]ll my teachers know that I am diabetic’ (13, F) and Aiden added that: ‘[*T*]he school helps me’ (13, M) and further clarified that at school, ‘It’s my teachers’ (13, M) who assists him.

Participants also received support through mentoring from experts and others living with T1D. Ivy said, ‘[*U*]h my doctors are really amazing and whenever we have issues, we can just message them or phone them and they’re always on standby’ (15, F). Liam added that positive reinforcement is beneficial in self-managing: ‘Also as I go on there are some people who says, “[*O*]h you did good, great job continue with that”’ (14, M). Participants also mentioned the support from others living with T1D as helpful.

### Theme 3: The silver lining of developing positive characteristics

Adolescents with T1D frequently acquire advantageous attributes as a result of controlling their medical condition. It can be daunting to self-manage and keep a well-controlled T1D. However, this is mitigated by the adolescent’s ability to persevere and overcome obstacles.

Participants noted that self-managing T1D and keeping it well-controlled leads to personal growth. Participants indicated that after the diagnosis, they developed positive characteristics they had never experienced before. John expressed: ‘So it was tough to figure that out. But I did get there’ (17, M). Ivy indicated that she developed a greater sense of responsibility: ‘I wanted the independence’ (15, F). Similarly, Liam mentioned: ‘I don’t know but [*this*] is all a personal mindset that I have grown’ (14, M), and Inca adds: ‘I think it’s just made me confident’ (14, M).

Most participants expressed determination, drive and commitment to achieving their goals and effectively controlling their diabetes. Liam indicated: ‘I wanna accomplish something … I feel happy because I know I did a good job with controlling it’ (14, M). John also noted that recognising accomplishment is sound reinforcement: ‘It’s like, oh okay, that’s what I do, and I see that it actually works. So, I must be doing something correct’ (17, M).

### Theme 4: It is a process of continuously learning about type 1 diabetes

Participants highlighted the fact that seeking information to educate themselves about self-managing or keeping T1D controlled contributed to their ability to maintain a well-controlled T1D. Participants reported that increased bodily awareness helped them understand their bodies better. John and Ivy mentioned that knowing your body helps a lot. Similarly, Nathan mentioned that he senses when he requires correcting: ‘[*S*]ay if my blood sugar is low I would feel it and then I want to go check it’ (12, M). Participants admitted that gaining knowledge of their bodies and becoming more aware of their responses required trial and error, which involved some learning. Liam expressed: ‘[*I*]t was quite confusing when I first started’ (14, M). Liam said he had to: ‘[*S*]et up a plan to work all of it out’ (14, M). Inca explained that after a while, ‘[*I*]t’s kind of just … knowing what my body does now’ (12, F).

Participants mentioned that they researched diabetes from various sources to gain more knowledge on diabetes management. They asked for guidance on self-managing T1D from their healthcare team and online sources, including social media. Additionally, many participants relied on mobile apps, wearable devices and other technologies to monitor their blood sugar levels, dietary intake and physical activity. The participants indicated that, with the healthcare team’s assistance, they could gather more information beyond what was being taught by the doctor. Participants expressed that acquiring the skills and knowledge required to successfully self-manage their condition was also rewarding. Ivy highlighted that seeking out information was more beneficial: ‘[*W*]anting to do it myself and wanting to learn more about it myself and not being completely reliant on other people to look after me’ (15, F). And John expressed: ‘I’ve had to do a lot of stuff by myself and I’ve actually found it rewarding to figure it out’ (17, M).

In summary, Table 2 showcases the themes and subthemes, and the implications thereof, further in the text:

**TABLE 2:** Summary of protective factors that promote resilience and support type 1 diabetes self-management in adolescents receiving private healthcare in South Africa.

Theme	Protective factors identified	Implications for practice
1. Just do it: Commit to the diabetes care plan	Planning and being prepared Staying active Their responsibility Perseverance	Encourage the development of structured routines, goal-setting, and self-management tools in adolescent care plans.
2. It takes a village to raise a child living with diabetes	Parents and other family members Peer support Support from the school Mentoring from experts and others living with T1D	Promote collaborative care involving families, schools, and peer networks; improve access to diabetes mentorship.
3. The silver lining of developing positive characteristics	Personal growth Determination	Integrate psychosocial support that fosters autonomy, goal achievement, and identity development.
4. It’s a process of continuously learning about T1D	Bodily awareness Gaining more knowledge	Facilitate access to diabetes education, digital tools, and continuous learning opportunities.

T1D, type 1 diabetes.

## Discussion

This study aimed to explore and identify the protective factors of resilience among adolescents living with well-controlled T1D. The data were consistent with the definition of resilience in that adolescents with well-controlled T1D had positive outcomes and successfully managed their T1D. This task can be challenging and demanding, especially for adolescents transitioning from childhood to adulthood. However, our findings from this research indicate that protective factors of resilience may contribute to self-management and maintaining well-controlled T1D. The data confirmed that protective factors of resilience can enhance adolescents’ resilience and help those with T1D develop the ability to self-manage their diabetes effectively. The key findings and their relationship to previous research or existing knowledge, practice and policy are discussed further in the text.

### Internal abilities

The participants demonstrated various internal resilience abilities in this research, which promoted resilience and helped maintain well-controlled T1D. In the first theme, ‘Just do it: Commit to the diabetes care plan’, adolescents demonstrated an ability to prepare and plan, taking responsibility and showing perseverance. The determination was evident in the third theme, The silver lining of developing positive characteristics. To continuously self-manage T1D, all participants emphasised the importance of perseverance and facing the challenges of managing T1D head-on.^41^ Their sense of capability and competence made taking ownership of their T1D and maintaining this responsibility successful over time.^41^ The ability to plan and be organised has helped our participants manage their well-controlled T1D. These findings support Vollrath et al.^42^ who highlighted the importance of adolescents living with T1D being organised and planning to ensure they keep blood glucose within an acceptable range. The importance of being a planner for diabetes patients can lead to lasting positive outcomes in glucose control.^43^ Their determination is a key internal protective factor for adolescents to manage T1D better. Those who exhibit a strong drive and dedication towards achieving their goals may have an advantage in managing their diabetes effectively.^42^ They can create specific health objectives, like maintaining optimal blood sugar levels, and put in deliberate efforts towards meeting these objectives. This is consistent with the data in this study, as participants were able to plan, set goals and achieve them whilst making daily decisions and successfully managing T1D.

Willingness to learn was evident in the fourth theme; It is a process of continuously learning about T1D. Patient education is essential to the management of T1D.^44,45^ Studies have shown that managing T1D requires ongoing effort and self-awareness.^45^ To optimise outcomes and minimise risks associated with this condition, patient education is necessary. Individuals with an in-depth understanding of T1D are better equipped to manage their glucose levels, prevent complications and select suitable treatments for themselves.^45^ This study demonstrated that adolescents with well-controlled T1D acquired the skills and knowledge required to successfully manage their condition by learning from various sources and applying these skills to their daily routines.

### External resources

External protective factors refer to environmental resources that promote resilience.^46^ Theme 2: It takes a village to raise a child living with diabetes, highlighted the various external resources such as family support, healthcare team support, peer support groups, school support and the advancement of technology and social media. Participants benefitted from the extensive environmental resources that contributed to managing their medical conditions and overall wellness.^46^

In our study, we found that for adolescents living with T1D, the support of family members is crucial in managing their medical condition. By supporting healthy dietary practices, encouraging regular physical activity, assisting with insulin administration and providing emotional support, families can help create a supportive home environment. Families play an integral role in encouraging and providing support to adolescents living with T1D. It was evident from the data that family support and encouragement remained prevalent throughout early to late adolescence, albeit with varying degrees of assistance. The family’s involvement in practicing healthy eating habits can also foster a supportive environment, thus decreasing feelings of isolation.

The support of the healthcare team plays a crucial role in managing T1D. Building a strong relationship with the healthcare team is essential. Participants mentioned the major role the endocrinologist played in ensuring adolescents kept well-controlled T1D.

Another integral part of external resources as protective factors are peer support groups and support at school. Peer support groups are beneficial because adolescents with T1D can benefit from interacting with peers who are also living with T1D. Peer support groups provide individuals with a platform to discuss their experiences, receive advice and guidance from others with the same condition and access emotional support. Through regional diabetes associations, internet forums or social media sites, one can locate these groups. Salis et al.^47^ mentioned that adolescents in their research indicated that having peer support groups made self-managing T1D less burdensome. Additionally, because adolescents spend a significant amount of time in school, teachers and the school must create learning environments that understand and accommodate adolescents with T1D.^47^ This was consistent in the data of this study; teachers created an environment that allowed adolescents the freedom to correct themselves, whether during class or mealtime, to handle any potential issues that might arise during school hours.

The most recent external resource as a protective factor is the advancement of technology and social media. The role of social media in acquiring information among peer support groups has increased tremendously. The use of technology to facilitate communication has increased social and emotional support, as well as self-management of T1D. Salis et al.^47^ noted that social media platforms, such as Facebook, YouTube and Instagram, have increased information sharing. This was evident in the current study, as it improved companionship and group cohesion.

### Process of positive adjustment

The internal abilities and external resources enabled these adolescents living with T1D to adjust well to the management behaviour tasks, leading to living with well-controlled T1D, and also facilitated personal growth, as evident in theme 3: The silver lining of developing positive characteristics. This aligns with Masten’s^8^ theory, highlighting the fact that protective factors play a role in determining a person’s capacity to respond favourably to significant adversity. In this study, social competence, autonomy and a sense of self^48^ were represented in planning, staying active, taking responsibility for managing their condition and being determined to master the management whilst continuously learning and becoming more aware. Masten^49^ further highlighted the importance of effective caregivers and meaningful connections to other competent and caring adults. This is evident in the roles played by parents, other family members and medical professionals in the lives of individuals living with well-controlled T1D. Other fundamental resources that Masten^48^ highlighted include pro-social peers, friends, effective teachers and schools, all of which play an invaluable role in supporting resilience. These internal abilities and external resources served as protective factors, helping adolescents with T1D overcome the adversity of living with the condition and report personal growth, improved well-being and a healthy, fulfilling life.

### Strengths and limitations

This study offers valuable insight into how adolescents with T1D, who access private healthcare in South Africa, manage their condition successfully. A key strength is that it focuses on young people during a significant life transition – moving from childhood to adulthood – when managing a chronic condition like T1D can be especially challenging. By hearing directly from adolescents who have kept their diabetes well-controlled, the study provides a clear and grounded understanding of what helps them cope and stay healthy. It highlights the personal strengths they draw upon, such as being prepared, responsible and open to learning, as well as the support they receive from others, including family, friends, teachers and healthcare providers. These insights provide valuable guidance for healthcare professionals and families supporting adolescents with T1D. The findings can help reinforce what is already working well and suggest ways to better support those who are struggling with self-management.

One of the significant limitations of this study is that it included only seven participants, drawn from a privileged cohort with access to educational resources and an expert team of medical professionals, which may have afforded them better access to healthcare resources, support systems or educational interventions. These participants were already able to manage their T1D effectively. They had to have access to connectivity and devices, as data generation took place online, because of stricter regulations resulting from the COVID-19 pandemic. Adolescents receiving care in public health settings, where resources are often more limited and systemic challenges more prevalent, might experience or articulate resilience differently. Even though the sample size is so small, and the findings are not generalisable to a larger cohort of people who are managing their diabetes well, this study provides valuable insight into diabetes management behaviours and is one of only a few in South Africa that gives direction to new research that could be conducted within this context.

### Implications and recommendations for future research

This study provides a foundation for further exploration of adolescent internal abilities and external resources in South Africa, and potentially elsewhere, that facilitate the effective management of T1D. For instance, this study could be replicated in other provinces in South Africa or with similar cohorts of privileged individuals to determine whether internal abilities and external resources differ based on geographical area or location. The same could apply to groups of the same age but from different socioeconomic backgrounds that receive their treatment through public healthcare facilities. A longitudinal study could track these cohorts to see if they continue to manage their diabetes effectively during and after adolescence, and if the behavioural changes and diabetes care management process are learned and incorporated habitually into their everyday lives as adults. Future research should explore the role of contextual factors, including disparities in healthcare access and quality, in shaping the resilience processes of adolescents living with T1D.

## Conclusion

Self-management of T1D poses challenges that can vary by age and stage of life. Our study focused on adolescents transitioning from childhood to adulthood. It identified four protective factors of resilience that our sample of adolescents living with T1D experienced, which assisted them in the successful self-management of their condition. They highlighted the protective factors that continuously assisted them with self-managing their medical condition. Their internal abilities, such as preparing and planning, taking responsibility, persevering, being determined, engaging in personal growth and being willing to learn, helped them manage T1D effectively. These internal abilities are innate traits; however, they can be enhanced and promoted through the experience of adversity. Furthermore, participants highlighted external resources as protective factors that helped them manage their T1D effectively. These included family support, healthcare teams, peer support groups, school peers and teachers, as well as advancements in technology. The involvement of external resources in self-managing T1D helps adolescents perceive the management of T1D as manageable and reduces feelings of isolation.

This research study explored protective factors that fostered resilience and supported effective self-management of T1D among adolescents accessing private healthcare services in South Africa. Given the distinct context of private healthcare, these findings may not fully reflect the experiences of adolescents in public health settings, where structural challenges and resource constraints may influence the articulation and development of resilience differently. As such, the transferability of these findings is limited to similar, well-resourced contexts. However, the potential for adolescents with T1D to utilise available internal abilities and external resources in response to significant adversity in their lives can benefit the successful management of T1D. As mentioned earlier in the text, the higher the resilience, the lower the vulnerability and maladaptive outcomes to significant threats. Protective factors will help promote health and alleviate the impact of the condition in the event of illness. This study could further inform practitioners on how to support adolescents who manage their diabetes effectively, helping them continue to do so, and vice versa. It could also aid those who are not managing it well, providing insight into what they can do differently to manage the condition more effectively.
